# Machine learning of metabolite–protein interactions from model-derived metabolic phenotypes

**DOI:** 10.1093/nargab/lqae114

**Published:** 2024-09-03

**Authors:** Mahdis Habibpour, Zahra Razaghi-Moghadam, Zoran Nikoloski

**Affiliations:** Bioinformatics Department, Institute of Biochemistry and Biology, University of Potsdam, Potsdam, Germany; Bioinformatics Department, Institute of Biochemistry and Biology, University of Potsdam, Potsdam, Germany; Systems Biology and Mathematical Modeling, Max Planck Institute of Molecular Plant Physiology, Potsdam, Germany; Bioinformatics Department, Institute of Biochemistry and Biology, University of Potsdam, Potsdam, Germany; Systems Biology and Mathematical Modeling, Max Planck Institute of Molecular Plant Physiology, Potsdam, Germany

## Abstract

Unraveling metabolite–protein interactions is key to identifying the mechanisms by which metabolism affects the function of other cellular layers. Despite extensive experimental and computational efforts to identify the regulatory roles of metabolites in interaction with proteins, it remains challenging to achieve a genome-scale coverage of these interactions. Here, we leverage established gold standards for metabolite–protein interactions to train supervised classifiers using features derived from genome-scale metabolic models and matched data on protein abundance and reaction fluxes to distinguish interacting from non-interacting pairs. Through a comprehensive comparative study, we explore the impact of different features and assess the effect of gold standards for non-interacting pairs on the performance of the classifiers. Using data sets from *Escherichia coli* and *Saccharomyces cerevisiae*, we demonstrate that the features constructed by integrating fluxomic and proteomic data with metabolic phenotypes predicted from genome-scale metabolic models can be effectively used to train classifiers, accurately predicting metabolite–protein interactions in the context of metabolism. Our results reveal that the high performance of classifiers trained on these features is unaffected by the method used to generate gold standards for non-interacting pairs. Overall, our study introduces valuable features that improve the performance of identifying metabolite–protein interactions in the context of metabolism.

## Introduction

Metabolite–protein interactions (MPIs) represent just one type of interactions between biochemical components in cell systems, with important implications on activity of individual proteins and entire pathways (1–4). While there are diverse experimental approaches that can help in elucidating MPIs (5–9), they have helped in cataloguing just a fraction of all possible MPIs, gathered in different databases (e.g. STRING (10) and PMI-DB (11)). The resulting insights have provided a sufficient basis for developing machine and deep learning models for prediction of MPIs based on protein and metabolite structural features (12–16). However, these experimental and *in silico* approaches remain silent regarding the functional relevance of the elucidated MPIs.

Recently, we reviewed and compared computational approaches for prediction of MPIs using features obtained from constrained-based metabolic modeling (17). Constraint-based modeling approaches make use of genome-scale metabolic models (GEMs) to obtain features for reactions, catalyzed by particular proteins, which together with features for metabolites are employed to train machine and deep learning models to identify MPIs (18–21). In this setting, fluxes represent reaction features that are generated either by applying metabolic flux analysis with metabolite labeling patterns (22–24) or by using computational approaches from the constraint-based modeling framework (25).

The flux, $v$, of a reaction catalyzed by a single enzyme depends on the turnover of the enzyme, ${{k}_{cat}}$, the enzyme abundance, $E$, and a function $\eta ( \cdot )$ of parameters, ${{\bf k}}$, concentrations of metabolites, ${{\bf x}}$, acting as substrates or regulators, i.e. $v = {{k}_{cat}}E\eta ( {{{\bf x}},{{\bf k}}} )$. The function $\eta$ captures the effect of MPIs on reaction fluxes, and is determined by the enzyme kinetics describing the reaction mechanism. The latter involves multiple parameters, ${{\bf k}}$, and non-linear dependences, rendering it difficult to directly model $\eta$ in terms of metabolite concentrations. This problem is that more challenging since enzyme kinetics differ between reactions steps in a metabolic network. For instance, in the case of Michaelis–Menten-like kinetics without regulation, $\eta ( {{{\bf x}},{{\bf k}}} ) = \frac{{\mathop \prod \nolimits_j {{{( {\frac{{{{x}_j}}}{{{{K}_{m,j}}}}} )}}^{{{\alpha }_j}}}}}{{1 + \mathop \prod \nolimits_j {{{( {\frac{{{{x}_j}}}{{{{K}_{m,j}}}}} )}}^{{{\alpha }_j}}}}}$, with ${{\alpha }_j}$ denoting the stoichiometry with which a metabolite enters as a substrate and ${{K}_{m,j}}$ denoting the respective Michaelis constant. Note that this form implies that increases in the concentration of substrates, that bind to the enzymes and represent a typical form of MPI, leads to increases in the value of $\eta$ and the corresponding flux. Consideration of metabolites that bind to enzymes and lead to its activation or inhibition without being modified by the enzyme action can be captured by modifying the mentioned form of $\eta$ to include multiplication factors (e.g. as in the case of the convenience kinetics (21).

In contrast to fluxes, which can be predicted using approaches from the constraint-based modeling framework (26), complete data on metabolite concentrations, providing full coverage of the studied cell system, are more difficult to obtain (27). This is largely due to the need to use diverse metabolomics technologies to quantify the entirety of different compound classes (21). As a result, constraint-based modeling has been used to derive proxies for metabolite concentrations, such as: shadow prices (28), metabolite turnover (29) and flux sums (30). Shadow prices and metabolite turnovers have been used to predict MPIs (19), (29), however without considering their effects on $\eta$. Inferring the latter requires access to matched fluxomics and quantitative proteomics data sets, which have recently become available for *Escherichia coli* (31), *Saccharomyces cerevisiae* (32), *Chlamydomonas reinhardtii* (33) and *Arabidopsis thaliana* (34). Recent comparative analysis further ranked the performance of these approaches based on the usage of shadow prices and metabolite turnovers predicted based on metabolic models and constraint-based approaches (35), demonstrating their added value in comparison to approaches that rely on features derived from protein sequence and metabolite structure (12).

Here we make use of the available matched fluxomics and proteomics data sets for *E. coli* and *S. cerevisiae* along with available gold standards of MPIs for these model organisms to train organism-specific classifiers that can accurately distinguish interacting from non-interacting metabolite–protein pairs. The organism-specific classifiers are based on features obtained from integration of these fluxomics and proteomics data, namely, flux sums, as proxies of metabolite concentrations, and $\eta$ values for reactions. In such a way, we circumvent the dependence of $\eta$ on multiple parameters, and only rely on proxies for metabolite concentrations. Our extensive analysis demonstrates that the supervised classifiers show excellent performance, which is robust to different strategies for selecting the gold standard for non-interacting metabolite–protein pairs.

## Materials and methods

### Data

Predicting MPIs using $\eta$ values of reactions and flux sums of metabolites relies on the availability of fluxomic and proteomic data. We made use of proteomic data across diverse environments, along with essential information on growth rates and mutations used in the later computational steps for flux estimation. To this end, we employed the data sets from two studies on *E. coli*, namely Heckmann *et al.* (31) and Davidi *et al.* (36), and the study on *S. cerevisiae* by Chen *et al.* (37).

The Heckmann data set collected proteomics data from four studies (38–41), containing protein abundance for 19 strains with different mutations (i.e. knocked-out genes). The Davidi data set gathered the protein abundance data from three studies (42–44) across 31 growth conditions corresponding to different carbon sources (e.g. glucose, galactose, acetate, pyruvate, fructose, fumarate, glucosamine, glycerol, mannose, succinate and xylose). For *S. cerevisiae*, the Chen data set provided proteomic data and estimated the flux distributions across 26 different conditions, utilizing information from four studies (45–48). These data sets allowed us to access a diverse set of conditions and genetic perturbations, improving the robustness of our predictions regarding MPIs.

Two gold standards, STITCH (49) and PMI-DB (11), were used to obtain MPIs in *E. coli* and *S. cerevisiae*. These benchmarks served as the basis for training and testing the machine learning classifiers. The PMI-DB database contains positive and negative labels for 34 359 metabolite–protein pairs in *E. coli*, with 1679 positive and 32681 negative instances. Further, STITCH provides confidence scores for MPIs, covering over 2 million pairs for *E. coli* and 3 million pairs for *S. cerevisiae*. Considering interactions with confidence scores exceeding 500 as positives in the STITCH database resulted in 206 817 positive labels for *E. coli* and 332 226 positive labels for *S. cerevisiae*.

### Defining the sets of positive and negative instances

In this study, we utilized two databases, the PMI-DB database, which contains interacting and non-interacting metabolite–protein pairs in *E. coli*, and the STITCH database, which contains confidence scores for MPIs in both *E. coli* and *S. cerevisiae*. Positive and negative labels can be obtained directly from the PMI-DB database; however, there is a lack of evidence for non-interacting metabolite–protein pairs (i.e. negative instances) in the STITCH database. To this end, we used three approaches to construct a negative set based on the STITCH database.

To establish the negative instances using an approach adopted from (50), hereafter referred to as *potential negative labeling*, all metabolite–protein pairs with a confidence score greater than 500 in the STITCH database were given a label of one, denoting interaction (i.e. positive set); otherwise, their label was assumed to be zero, denoting lack of interaction (i.e. assumed negative set). We employed several SVM classifiers to score pairs in the assumed negative set to predict potential negatives. First, we partitioned the set of assumed negatives into $t$ subsets, with $t$ denoting the ratio of the total number of instances in the assumed negative set to the number of instances in the positive set$.$ In each iteration, the set of metabolite–protein pairs with label one was used along with one of the $t$ subsets with assumed label zero, which by construction are of the same size, to train the SVM model, predicting the class of the remaining pairs. In this process, $t$ SVM classifiers were trained, and the prediction results of applying each classifier to the metabolite–protein pairs in the remaining $t - 1$ subsets were stored. We then computed a score for each metabolite–protein pair in the assumed negative set by counting the SVMs that predicted it as a negative. With $t$ classifiers, the minimum possible score for each pair is zero and the maximum is $t - 1$. The pairs with a score of $t - 1$, indicating that they were consistently predicted as negative in all iterations, were in turn selected as potential negative instances.

Alternatively, to build the negative instances based on an approach from (12), termed *random STITCH labeling*, for each data set, metabolite–protein pairs present in the STITCH database with a confidence score above 500 were treated as part of the positive set. For the remaining pairs, which either have a lower STITCH confidence score or are not presented in STITCH, if both the metabolite and the protein in the pair were found in the positive set, the pair was labeled as negative.

In the third approach, termed *Tanimoto labeling*, the negative set is determined based on the Tanimoto similarity (51) between the 64-bit fingerprints of the metabolites. For each pair of a metabolite $Me{{t}_i}$ and a protein $Pro{{t}_j}$ in the STITCH database with a confidence score above 500, the fingerprint similarity between $Me{{t}_i}$ and all other metabolites ($Me{{t}_{i^{\prime}}},\ i^{\prime} \ne i$) were calculated using the Tanimoto similarity; for the metabolite $Me{{t}_{i^{\prime}}}$ with zero similarity to $Me{{t}_i}$, if the pair $Me{{t}_{i^{\prime}}}$ and $Pro{{t}_j}$ was not present in the positive set, this pair was considered as non-interacting (i.e. belongs to the negative set).

To assess the performance of our features with different strategies for constructing the class of negative instances, we implemented random labeling, where each pair was randomly assigned a negative or positive label while maintaining the balance between two classes, and compared the results with our adopted strategies.

### Feature from matched fluxomics and proteomics data sets combined with metabolic modeling

For each of the two *E. coli* data sets considered (see Materials and methods), the flux distributions were estimated using parsimonious flux balance analysis (pFBA) (52). This was achieved by considering the information on growth rates, uptake fluxes, and mutations provided in the data sets. In addition, for the Heckmann data set, the measured upper bound and lower bounds of reaction fluxes, estimated by metabolic flux analysis, were used as bounds in pFBA. In the case of *S. cerevisiae*, flux estimates from Chen *et al.* (36) were used.

The flux sum, ${{M}_{k,j}}$, of metabolite $k$ in strain or condition $j$ was calculated by ${{M}_{k,j}} = \frac{1}{2}\mathop \sum \limits_r | {{{S}_{k,r}}} |.{{v}_{r,j}}$. Hence the flux sum features for metabolite $k$ is a vector of flux sums across all investigated conditions. In this calculation metabolites were grouped according to their cellular compartments. Consequently, for *E. coli*, flux sums were calculated separately for the cytoplasm and periplasm compartments over all metabolites in the different experiments. For *S. cerevisiae*, the flux sums were calculated for five compartments, namely: cytoplasm, peroxisome, mitochondrion, nucleus, and endoplasmic reticulum.

**Figure 1.**
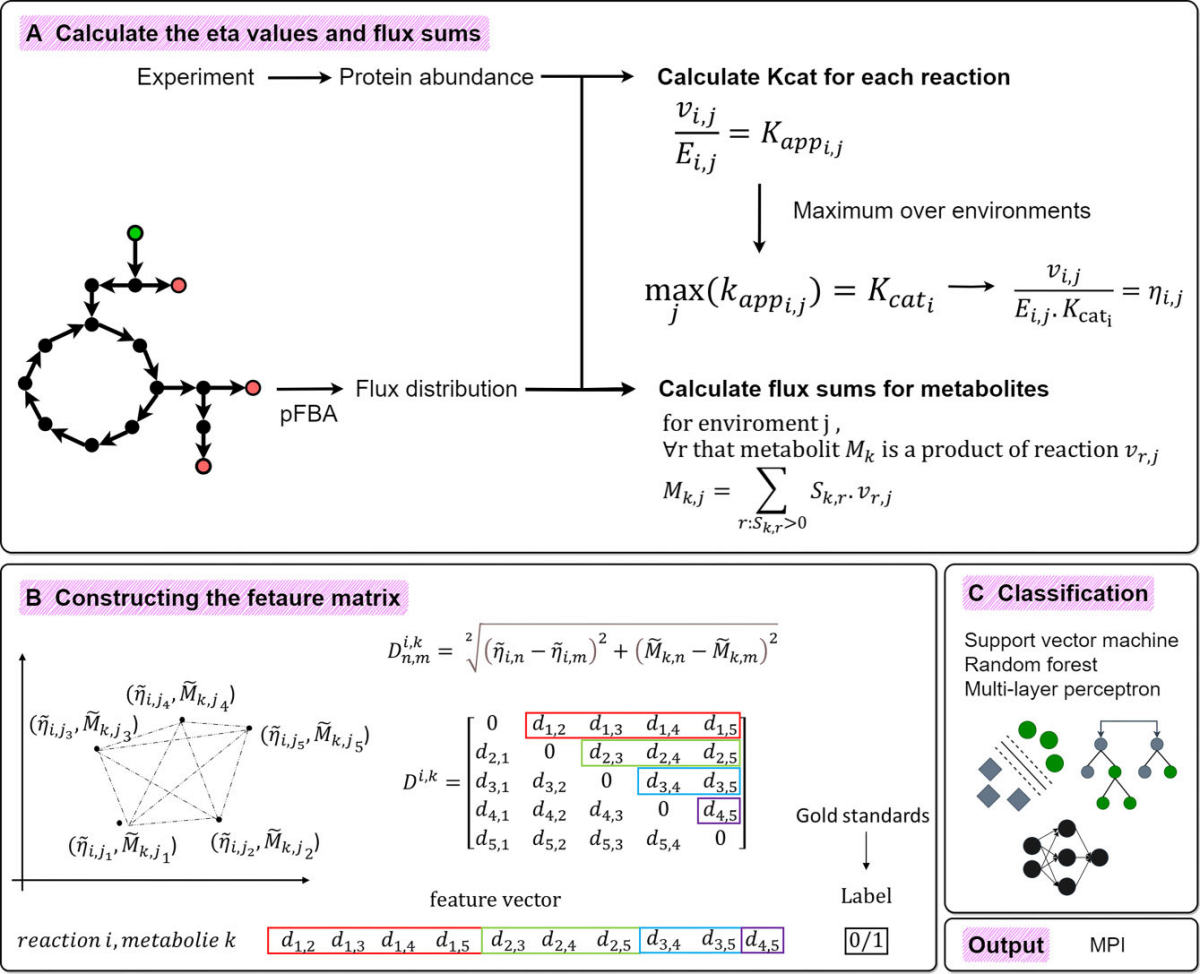
Overview of the approach for prediction of MPIs based on features from metabolic modeling. Summary of the computational steps to predict MPIs that include three main steps of calculating the ${\boldsymbol{\eta }}$ values for reaction and flux sums for metabolites, constructing the feature vectors using distance matrix and applying machine learning algorithms to classify metabolite-reaction pairs. ${\boldsymbol{v}}$: flux distribution, E: protein abundance, S: stochiometric matrix.

To obtain proxies for *in vivo*${{k}_{cat}}$ values, we followed the approach used in previously published works (31,35,36,53). We calculated the ${{k}_{ap{{p}_{i,j}}}}$ values by dividing the flux rate of reaction $i$ by the protein abundance of the enzyme catalyzing it for a given strain or condition $j$ (${{E}_{i,j}}$). For each reaction $i$, the ${{k}_{ca{{t}_i}}}$ was calculated such that ${{k}_{ca{{t}_i}}} = \mathop {\max }\limits_j ({{k}_{ap{{p}_{i,j}}}})$, where the maximum value of ${{k}_{ap{{p}_{i,j}}}}$ was selected across all strains or conditions. Finally, the $\eta$ value for reaction $i$ in strain or condition $j$ was calculated using ${{\eta }_{i,j}} = {\mathrm{\ }}\frac{{{{v}_{i,j}}}}{{{{E}_{i,j}}.{{k}_{ca{{t}_i}}}}}$ equation and the vector ${{\eta }_i}$ indicates the $\eta$ value for reaction $i$ across all conditions (Figure 1A).

The calculated $\eta$ and flux sum vectors were normalized across conditions for further steps, using the following normalization procedures: ${{\tilde{\eta }}_{i,j}} = \frac{{{{\eta }_{i,j}}}}{{\mathop {\max }\limits_j {{\eta }_{i,j}}}}$ and ${{\tilde{M}}_{k,j}} = \frac{{{{M}_{k,j}}}}{{\mathop {\max }\limits_j {{M}_{k,j}}}}$. After normalization, for each pair of $( {{{{\tilde{\eta }}}_i},{{{\tilde{M}}}_k}} )$, the coefficient of variation (CV) was calculated separately for $\tilde{\eta }$ values ($C{{V}_{{{\eta }_i}}}$) and normalized flux sums ($C{{V}_{{{M}_k}}}$) and the pair was removed from further analysis if either $C{{V}_{{{\eta }_i}}}$ or $C{{V}_{{{M}_k}}}$ obtained a value <1. This was performed because the machine learning models applied require variability of the independent variables used.

### Classification

We employed three machine learning models, Random Forest (RF), Support Vector Machine (SVM) and Multi-Layer Perceptron (MLP) for the classification of MPIs, as each model has its distinct characteristics and applications. RF, known for its accuracy, is an ensemble method that constructs multiple decision trees, introducing randomness to reduce overfitting. It is effective for large data sets with diverse features (54), and also provides a feature importance score that indicates the contribution of each input variable in predicting the output, aiding in understanding key contributing factors. SVM as a supervised learning algorithm seeks to identify a hyperplane to classify data by maximizing margins between data points (55). If the data is not linearly separable, SVM maps the input data points into a higher-dimensional space to find a linear decision boundary that separates different classes of data points. MLP is an artificial neural network designed for supervised learning (56). With input, hidden, and output layers, MLP employs backpropagation to adjust weights, enabling it to model complex relationships in data.

We performed fivefold cross-validation with 100 repetitions for all supervised approaches to ensure the robustness of our results and to obtain highly reliable performance measures. All the performance measures reported in this study are the averages taken over all repetitions. The dataset exhibits class imbalance, with significantly more negative than positive instances, potentially biasing the classifier towards negatives. To mitigate this problem, we ensured that each fold used to train the classifier contained an equal number of instances from both classes. This was achieved by including all positive instances in the selected training set (from the fivefold cross-validation) and randomly selecting an equal number of negative instances.

For the training process, we conducted grid-search for hyperparameter tuning across all models, and the parameters were fine-tuned through cross-validation over a parameter grid. The implementation of all models was carried out in Python using the Scikit-learn library. The entire code allowing reproduction of the findings is provided at https://github.com/mahdishb/MPI-ML.

## Results

### Classification of metabolite–protein pairs

To predict interacting or non-interacting metabolite–protein pairs, we used two novel features, $\eta$ values for each reaction and flux sums for each metabolite across the investigated environments (see Materials and methods). Since the $\eta$ values are associated to reactions, this resulted in a feature vector for each pair of a reaction and metabolite. The gene–protein–reaction (GPR) rules obtained from the metabolic model were then employed to connect each protein (enzyme) to the corresponding catalyzed reaction and transform metabolite–reaction pairs to metabolite–protein pairs.

To create the feature vectors, we applied a methodology inspired by the GRADIS approach (49) to create a distance matrix for each metabolite-reaction pair. To this end, for each reaction–metabolite pair, unique points were defined in the unit square with coordinates $( {{{{\tilde{\eta }}}_{i,j}},{{{\tilde{M}}}_{k,j}}} )$. Subsequently, for reaction $i$ and metabolite $k$, a distance matrix ${{D}^{i,k}}$ was constructed using the pairwise Euclidean distance between specified two points in environment $n$ and $m$ such that $D_{n,m}^{i,k} = \ \sqrt[2]{{{{{( {{{{\tilde{\eta }}}_{i,n}} - {{{\tilde{\eta }}}_{i,m}}} )}}^2} + {{{( {{{{\tilde{M}}}_{k,n}} - {{{\tilde{M}}}_{k,m}}} )}}^2}}}$. Finally, the rows of the upper right triangle of the ${{D}^{i,k}}$ were concatenated to form a feature vector for reaction $i$ and metabolite $k$. The above-mentioned process was repeated for all metabolite-reaction pairs resulting in a feature matrix used for training a machine learning model in later steps (Figure 1B and C).

Having formed the feature vectors, we employed three machine learning classifiers, namely, RF, SVM and MLP, to train classifier models for predicting MPIs (see Materials and Methods). We used four labeling strategies to label negative instances: (i) potential negative labeling, (ii) random STITCH labeling, (iii) Tanimoto labeling and (iv) PMI-DB based (see Materials and Methods). We note that the intersection of STITCH pairs with the Heckmann data set yielded 3305 positives and 204 304 unknown labels for metabolite–protein pair. Employing potential negative labeling strategy, 67081 pairs were identified as potential negatives. Similarly, the Davidi data set included 2429 positives and 139 499 unknowns, with 33 728 pairs identified as potential negatives. The Chen data set comprised 3364 positives and 188 400 unknowns, resulting in 67 747 potential negative pairs. Classifiers were trained only for the cytoplasm, since calculating the flux sums led to non-zero values only for the cytoplasm and mitochondrion; in addition, after filtering the pairs based on their coefficient of variation, there were no positive pairs left in the mitochondrion compartment.

**Figure 2.**
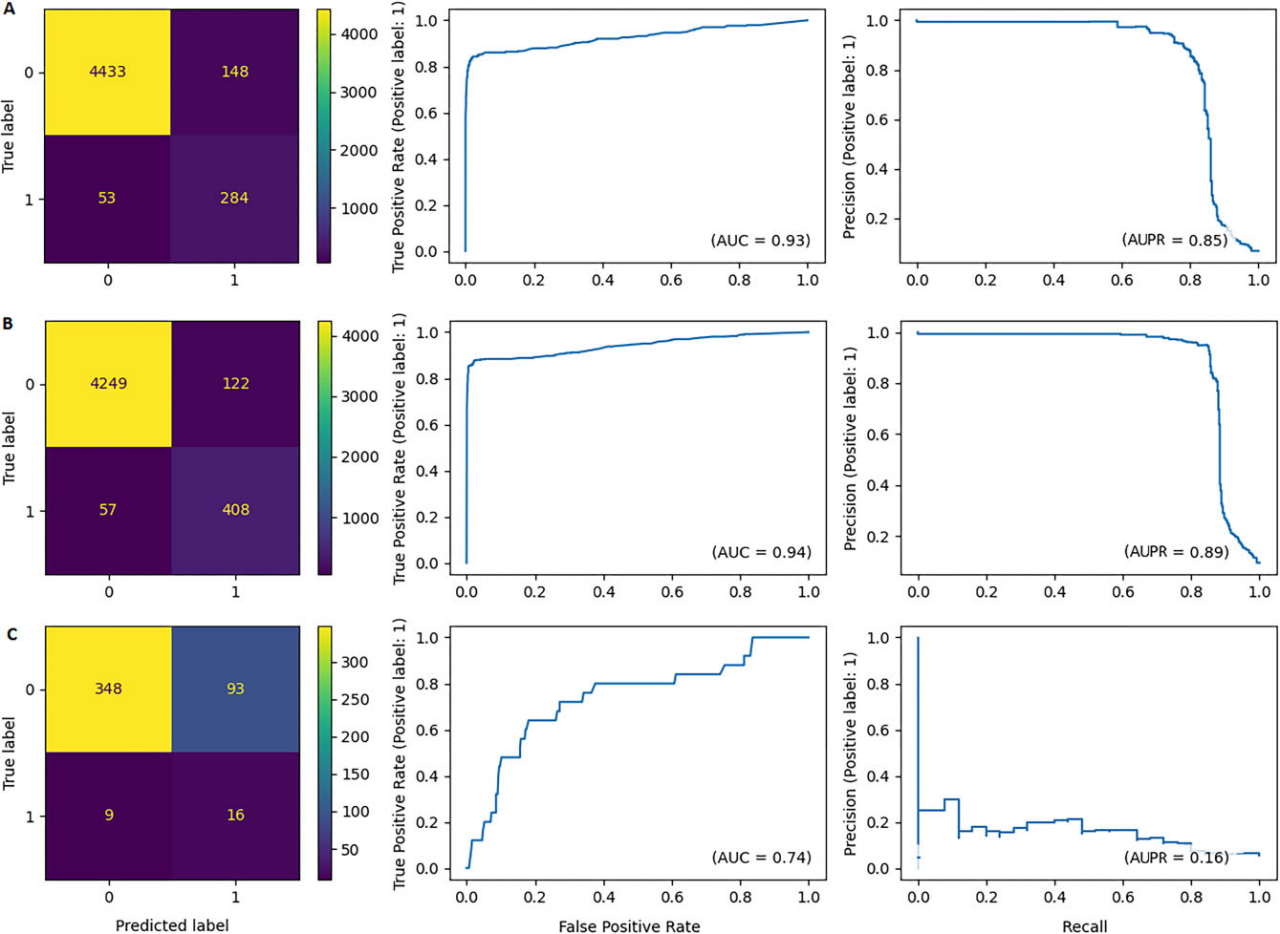
Performance of the RF classifier on three data sets using potential negative labeling strategy to label negative instances. The plots show the confusion matrices alongside the ROC and PR (Precision/Recall) curves for the classifiers trained with the featured obtained based on the (**A**) Davidi, (**B**) Heckmann and (**C**) Chen data sets. The plots also include the AUC and AUPR statistics.

**Figure 3.**
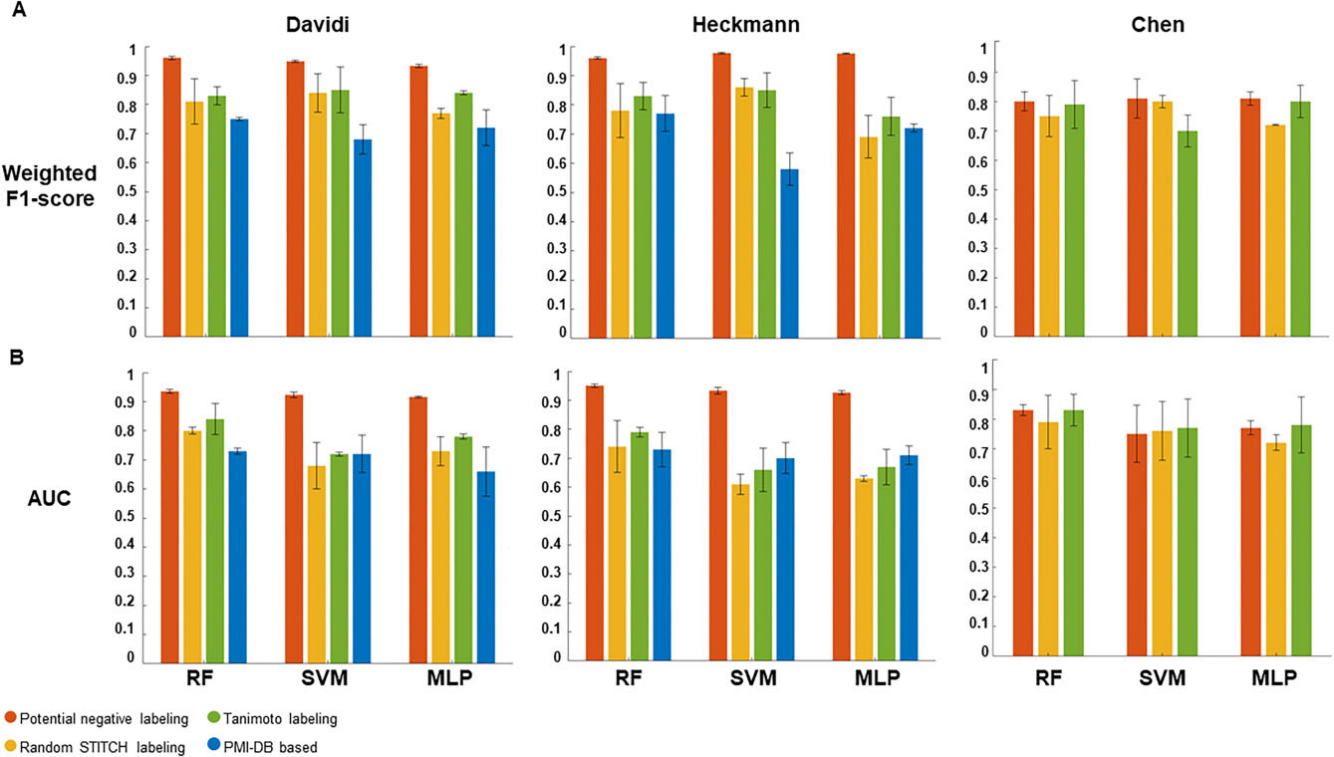
Performance of three classifier models using different negative labeling strategies. The plots in (**A**) and (**B**) show the performance of the three applied classifiers in terms of the weighted F1-score and AUC metrics, respectively, when different negative labeling strategies were used on the three data sets examined.

Using the potential negative labeling strategy on the STITCH database, the classifiers trained on the *E. coli* data sets showed the highest area under the receiver operating characteristic (ROC) curve (AUC) of 0.94 and 0.95 for the Davidi and Heckmann data sets, respectively, using the RF classifier (see Figures 2 and 3, and Supplementary Table S1). Performance for the other classifiers on these data can be found in Supplementary Figure S1 and Supplementary Figure S2. On the other hand, the RF classifier resulted in an AUC of 0.83 for *S. cerevisiae*. As shown in Figure 3 and Supplementary Table S1, the classifiers showed different performance with respect to other evaluation metrics. Notably, based on the weighted F1-score, RF demonstrated the best performance for the Davidi data set, while MLP outperformed for the Heckmann and Chen data sets. In the case of potential negative labeling strategy, the area under the precision/recall curve (AUPR) in the case of *E. coli* was the highest for the RF classifier trained with both the Davidi (0.86) and the Heckmann (0.89) data set (see Figures 2 and 3, Supplementary Table S1). In the case of *S. cerevisiae*, the AUPR was markedly lower for all classifiers, exhibiting the highest value of 0.28 for the RF classifier (Supplementary Table S1, Figure 2).

For the Davidi data set, using the random STITCH labeling 1.71% and 31.72% out of 141 928 metabolite–protein pairs were labeled as interacting and non-interacting pairs, respectively. For the Heckmann data set, out of 107 609 pairs, 3.07% were assigned positive and 69.3% were assigned negative labels. On the other hand, for the Chen data set, 1.75% and 39.67% out of 191 764 pairs were labeled with positive and negative labels, respectively. Figure 3 and Supplementary Table S1 illustrate that employing the random STITCH labeling strategy to label negative instances resulted in different classifier model performance across the data sets examined. For example, the SVM classifier exhibited the highest performance across all three data sets in terms of the weighted F1-score, while the opposite was observed based on the AUC metric. In addition, Supplementary Figure S3 illustrates the ROC and PR (Precision/Recall) curves when the RF classifier was applied to the three data sets using the random STITCH labeling strategy. The RF classifier showed the highest average AUC of the classifiers examined. In contrast to the potential negative labeling strategy, the AUPR were very small for both *E. coli* data sets and the three classifiers, reaching the highest values of 0.28 for the RF classifier on the Davidi data set (Supplementary Table S1 and Supplementary Figure S1). For the random SITICH labeling of negative instances, the SVM classifier showed the best AUPR of 0.48 for the SVM classifier in the case of *S. cerevisiae* (Supplementary Table S1). Performance for the other classifiers on these data can be found in Supplementary Figure S4 and Supplementary Figure S5.

Applying the Tanimoto labeling strategy, the Davidi data set yielded 2.26% of interacting and 94.46% of non-interacting labels. The Heckmann data set resulted in 2.15% of positive labels and 94.2% of negative labels, while the Chen data set comprised of 5.8% pairs assigned positive label and 89.77% of pairs assigned with negative labels. Consistent with previous observations, the use of the Tanimoto labeling strategy resulted in different levels of performance across the three examined data sets using different classifier models (see Figure 3 and Supplementary Table S1). Having labeled the negative instances using the Tanimoto labeling strategy, Supplementary Figure S6 shows the ROC and PR (Precision/Recall) curves when the RF classifier was applied to the three data sets. Consistent with the findings from other negative labeling strategies, the RF classifiers on average outperformed the other classifiers. Performance for the other classifiers on these data can be found in Supplementary Figures S7 and S8.

When using class labels from the PMI-DB database, which includes ground truth for negative pairs, the AUC value was 0.73 for the Davidi data set and 0.75 for the Heckmann data set, both using the RF classifier were achieved (see Figure 3 and Supplementary Table S1). The RF classifier also exhibited the highest weighted F1-score compared to the other classifier models. Like in the case of random STITCH labeling, the Tanimoto labeling as well as the PMI-DB-based labeling of negative instances yielded very small values for the AUPR across all data sets and classifiers (Supplementary Table S1).

### Performance based on random labeling

We implemented random labeling by assigning random labels to each pair and then trained the machine learning models. Analysis of the results for the cytoplasmic compartment for the Davidi and Chen data sets yielded a maximum AUC value of 0.5, while for the Heckmann data set, the maximum AUC was 0.51 (Supplementary Tables S2 and S3). This result highlights that for all three data sets, the randomization process resulted in significantly lower accuracy compared to other labeling strategies used, implying that the feature constructed by the distance matrix of $\eta$ values and flux sums contains effective information for learning classifiers that can predict MPIs.

### Robustness analysis based on usage of modified features

To evaluate the possibility of using only the $\eta$ values as features in the classifier without compromising performance, we investigated the effect of replacing $({{\tilde{M}}_{k,n}} - {{\tilde{M}}_{k,m}})$ with a constant value in the construction of the distance matrix. We then built different classifiers using all the four strategies for labeling negative instances to predict MPIs.

The highest achieved AUC was for the Davidi data set (0.91) and the the Heckmann data set (0.86) using RF and SVM classifiers, while most of the remaining scenarios resulted in an AUC score close to random classification (see Supplementary Table S4). We note that the AUPR values were consistently smaller than those with the classifier trained based on the distance matrix of $\eta$ values and flux sums. Therefore, these results indicate that the flux sums together with the $\eta$ values both contributed to the learning of the MPIs and cannot be replaced by constant values.

To assess the importance of the features derived from the distance matrix on the performance of the classifier models, we generated an alternative feature matrix by concatenating the ${{\tilde{\eta }}_i}$and ${{\tilde{M}}_k}$ vectors for each reaction $i$ and metabolite $k$. After constructing this concatenated feature matrix and employing different negative labeling strategies, the three different classifier modes were trained on the data sets examined. The highest AUC score was achieved by the RF classifier and with the potential negative labeling strategy for the Davidi data set with a value of 0.98 (Supplementary Table S5). Although the highest performance of the trained classifiers build on the concatenated features was higher than that of those build on the distance matrix, Supplementary Tables S1 and S5 show that on average, the classifiers built on the distance matrix performed slightly better based on most of the evaluation metrics. In particular, when considering the PMI-DB database, which includes ground truth for negative instances, the AUC values were on average higher when the classifiers were built on the distance matrix, presented above.

Furthermore, we analyzed the feature importance values obtained from the RF classifier built on the concatenated features (see Supplementary Figure S9). This analysis showed that using the concatenated features in both *E. coli* and *S. cerevisiae* with all negative labeling strategies, resulted in the $\eta$ values as the most important features in most of the scenarios considered. However, since the contribution of metabolite features is not as strong as that of reaction features, this approach might not effectively distinguish interacting from non-interacting metabolite–protein pairs.

### Ranking the metabolites based on participation in predicted interactions

After obtaining the results of class predictions for metabolite-reaction pairs, we further investigated the extent to which different metabolites participate in MPIs. To this end, we selected the metabolite–protein pairs predicted as interacting by the RF classifier; for each metabolite in this set, we then determined the number of distinct reactions that it is predicted to regulate via interaction to the underlying enzyme.

**Figure 4.**
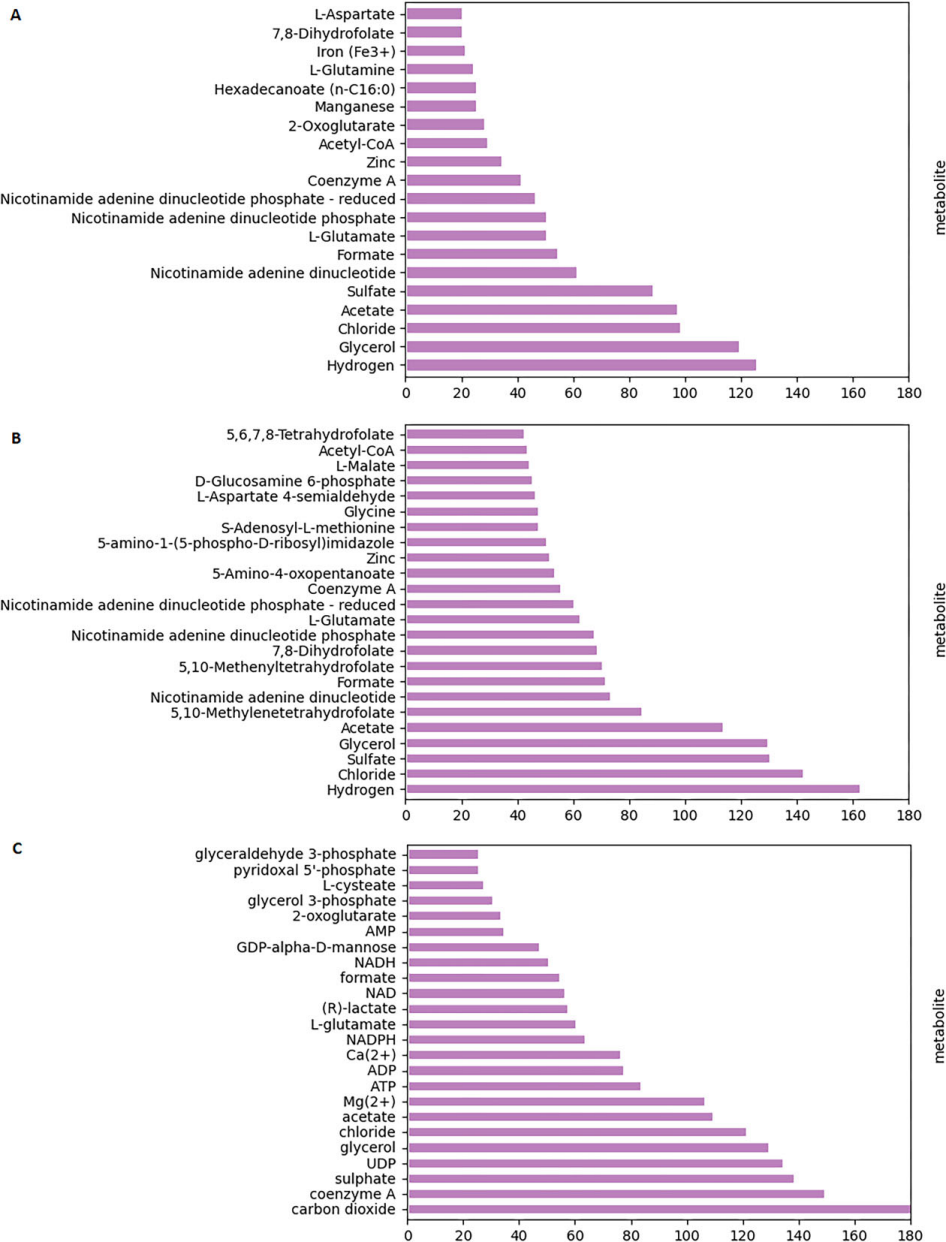
Ranking of metabolites based on the number of MPIs in which they are predicted to paricipate. The plots shows the metabolites alongside the number of MPIs in which there are predicted to be involved based on the classifiers for (**A**) Davidi, (**B**) Heckmann and (**C**) Chen data sets.

In the cytoplasm compartment of the Davidi data set, 340 metabolites contributed to the predicted MPIs, with 20 metabolites involved in over 20 distinct reactions. Hydrogen was the most interacting metabolite, predicted to affect 126 reactions, while glycerol ranked as second, affecting 120 reactions (Figure 4A). Hydrogen is known to affect metabolism via changes in pH, usually associated with enzyme inhibition (57). The cytoplasm compartment of the Heckmann data set included 380 metabolites with positive predicted reactions, with 36 metabolites contributing to more than 30 reactions. Here, too, hydrogen ranked first, affecting 163 reactions, followed by chloride with 143 reactions (Figure 4B). Exploring the cytoplasm compartment of the Chen data set revealed 372 metabolites predicted as MPIs, with 30 metabolites predicted to interact with protein affecting over 20 reactions. Carbon dioxide was the most interacting, affecting 188 reactions, followed by coenzyme A with 150 reactions (Figure 4C). Notably, chloride, glycerol, sulphate, acetate, coenzyme A, l-glutamate, nicotinamide adenine dinucleotide (NAD), and reduced nicotinamide adenine dinucleotide phosphate (NADPH) were present among frequently reported metabolites involved in MPIs in both *E. coli* and *S. cerevisiae*. The energy currency metabolites and cofactors are known to be among the most connected metabolites in metabolic models (58), in line with their principal role in modulating reaction fluxes.

## Discussion

Despite of the recognized and documented role of metabolites not only as substrates and products of metabolic reactions, but also regulators of enzyme activity, identification of MPIs and characterization of their effects on cellular phenotypes remains quite challenging. The identification of MPIs has been propelled by gathering data from different molecular profiling techniques (5–9) and using them as input in powerful machine learning approaches (12) to predict MPIs. However, the characterization of the role of MPIs lags behind due to the, often, numerous interactions in which particular metabolites are involved. Placing the MPIs in the context of large-scale metabolic network models has the potential to overcome this problem by relying on *in silico* investigations (17). To this end, the availability of matched fluxomics, proteomics, and metabolomics data have been instrumental in identifying MPIs and characterizing their role in metabolism (21). This is principally due to the fact the regulatory action of a metabolite is manifested on the effect its concentration has on the flux of reactions catalyzed by the interacting enzyme. The latter effect is captured by the enzyme kinetics, which mathematically describes how protein abundance and metabolite concentrations determine fluxes. However, following this approach necessitates assuming a particular mathematical form for enzyme kinetics as a function of protein abundance and metabolite concentration. This mathematical form involves multiple parameters whose estimation leads to non-linear optimization problems that are difficult to solve on a whole network level. The estimation of parameters also requires data on both fluxes as well as quantitative metabolomics data, which is further challenged by the need for a large coverage of metabolites for which quantitative metabolomics data must be gathered.

Here, we overcome this problem by casting the identification of MPIs as a classification problem, thus avoiding the need to assume a particular enzyme kinetics. In addition, by using proxies for metabolite concentrations, derived from constrained-based modeling, and the part of reaction fluxes directly and only affected by metabolite concentrations, obtained by combining proteomics data with flux estimates/predictions, we allow gathering full genome-scale coverage. The latter allows us to apply the classifiers to obtain better insights in the extent to which particular metabolites act as regulators of reaction flux.

To test our approach, we compared the performance of three classifier models trained with different gold standards of interacting and non-interacting metabolite–protein pairs. Of the three classifiers trained and based on each gold standard used, the RF classifier showed the highest average performance in terms of the AUC metric. RF classifiers also showed high AUPR values in the case of *E. coli* data sets. The use of the potential negative labeling strategy resulted in the highest average performance among the trained classifiers, which is probably related to its intrinsic training process. Interestingly, other strategies to assemble gold standards for non-interacting metabolite–protein pairs resulted in a comparable performance. This comparative analysis allowed us to demonstrate the robustness of the predicted MPIs with respect to the ways in which the non-interacting metabolite–protein pairs were determined.

However, the classifiers trained with omics data and metabolic model from one organism cannot be trivially generalized to another, since the features correspond to predictions from the metabolic model obtained by integration of data from particular experiments (with specified uptake/excretion fluxes, growth rates and protein abundances). Therefore, unlike classifiers of metabolite–protein interactions based on protein sequences and metabolite structure that can be applied across organisms (12), this is not the case the classifier in our study. However, we point out that MPIs predicted from these approaches are condition-independent. Therefore, despite the organism-specificity of the trained classifiers, we learn important insights into the functional relevance of MPIs via their effects on fluxes—which is a key benefit of the approach. Future directions that may boost the generalizability of models, developed based on omics data and metabolic models, across organisms would require setting-up experiments in which the investigated organisms are grown under same standardized conditions from which data are to be obtained.

Importantly, our approach builds on recent developments aimed at utilizing genome-scale metabolic models for engineering of features useful for accurate prediction of MPIs (19). These developments in the context of the constrained-based modeling framework pave the way for *in silico* characterization of the role of MPIs in metabolism.

## Supplementary Material

lqae114_Supplemental_Files

## Data Availability

The data underlying this article are available on GitHub under https://github.com/mahdishb/MPI-ML and https://doi.org/10.5281/zenodo.13341395. The data sets were derived from sources in the public domain: the STITCH database, http://stitch.embl.de/cgi/input.pl; the PMI-DB database, http://easybioai.com/PMIDB/download; and the BiGG Models, http://bigg.ucsd.edu. All code that was used to generate the results of this study, are available at GitHub (https://github.com/mahdishb/MPI-ML) and Zenodo (https://doi.org/10.5281/zenodo.13341395).
