# Supplementary material for: Machine learning of metabolite–protein interactions from model-derived metabolic phenotypes
Source: NAR Genom Bioinform. 2024 Sep 3;6(3):lqae114. doi: 10.1093/nargab/lqae114 (PMC11369697; doi:10.1093/nargab/lqae114)
Supplement: lqae114_Supplemental_Files [file lqae114_supplemental_files.zip › SupplementaryFigures.pdf]

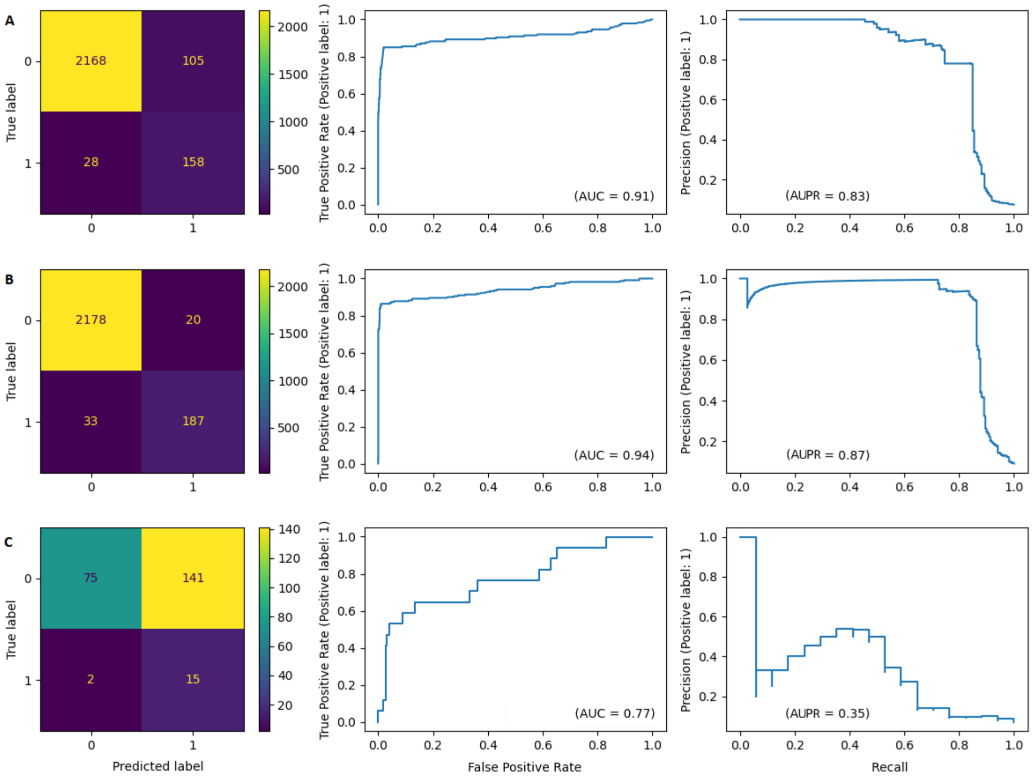

Figure S1. Performance of the SVM classifier on three data sets using potential negative labeling strategy to label negative instances. The plots show the confusion matrices alongside the ROC and PR (Precision/Recall) curves for the classifiers trained with the featured obtained based on the (A) Davidi, (B) Heckmann, and (C) Chen data sets. The plots also include the AUC and AUPR statistics.

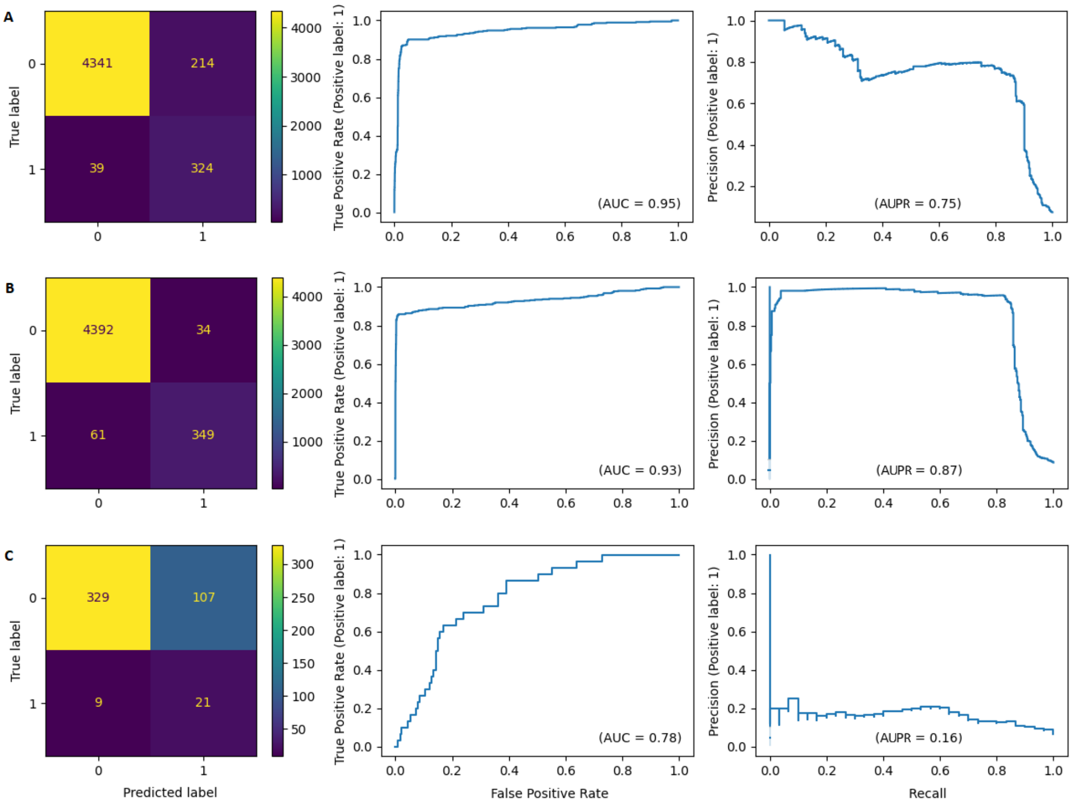

Figure S2. Performance of the MLP classifier on three data sets using potential negative labeling strategy to label negative instances. The plots show the confusion matrices alongside the ROC and PR (Precision/Recall) curves for the classifiers trained with the featured obtained based on the (A) Davidi, (B) Heckmann, and (C) Chen data sets. The plots also include the AUC and AUPR statistics.

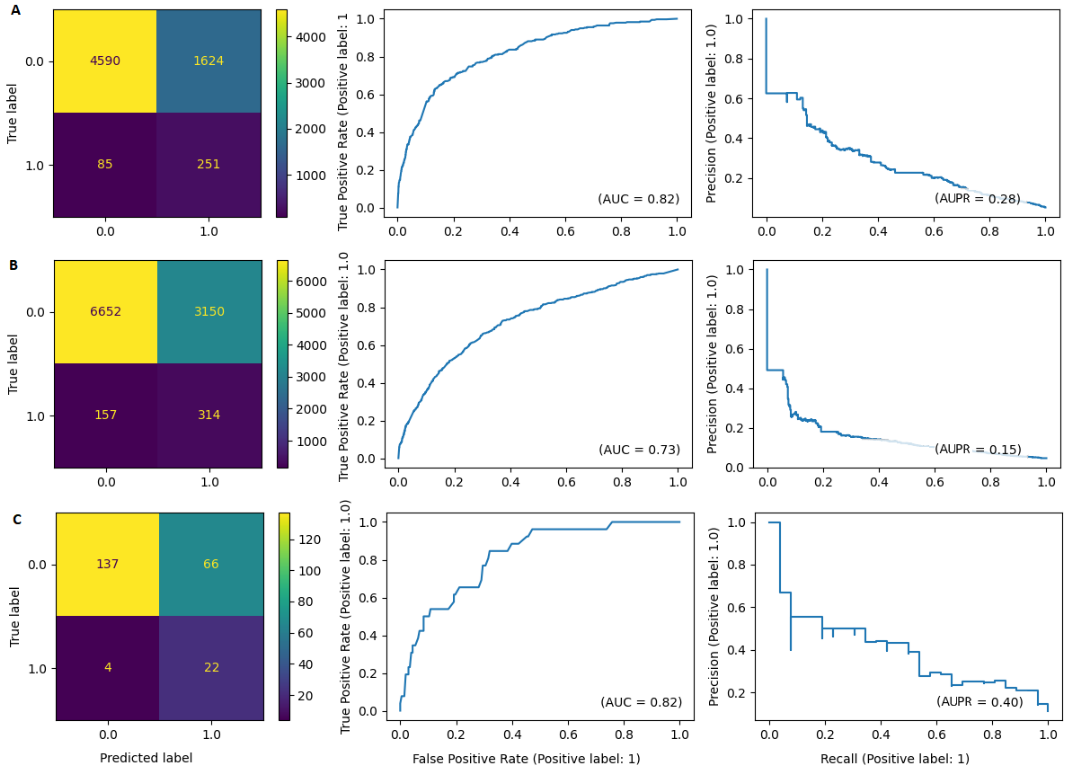

Figure S3. **Performance of the RF classifier on three data sets using the random STITCH labeling strategy to label negative instances**. The plots show the confusion matrices alongside the ROC and PR (Precision/Recall) curves for the classifiers trained with the featured obtained based on the (A) Davidi, (B) Heckmann, and (C) Chen data sets. The plots also include the AUC and AUPR statistics.

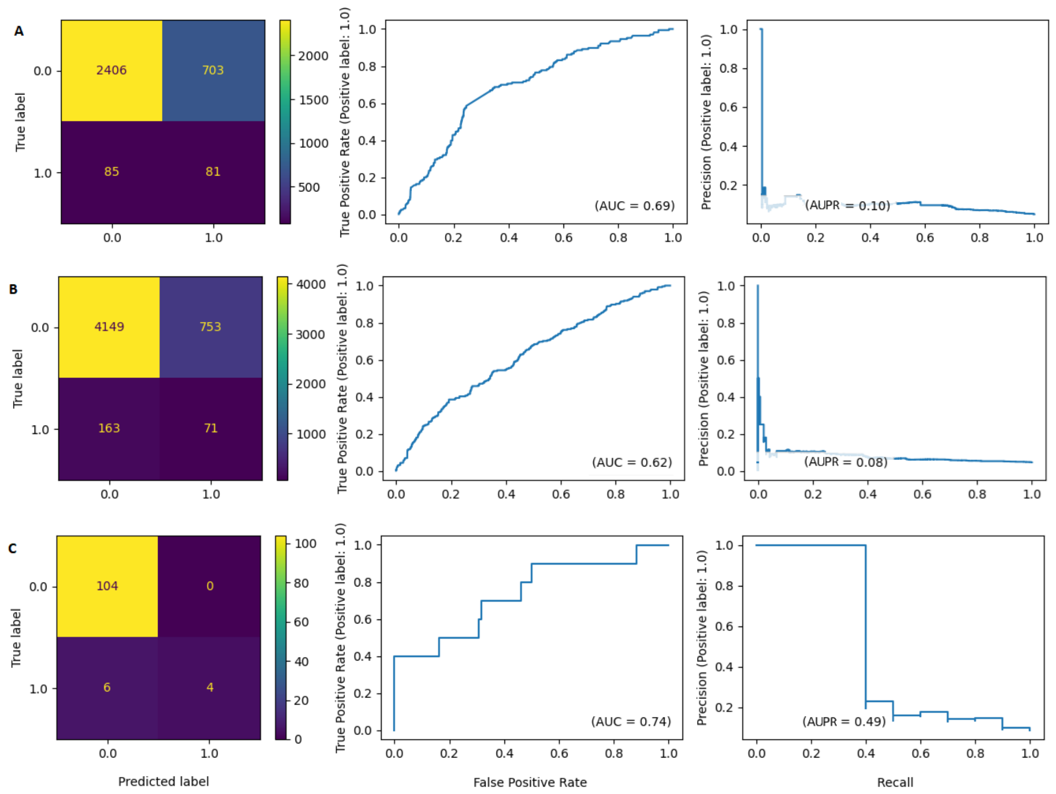

Figure S4. Performance of the SVM classifier on three data sets using the random STITCH labeling strategy to label negative instances. The plots show the confusion matrices alongside the ROC and PR (Precision/Recall) curves for the classifiers trained with the featured obtained based on the (A) Davidi, (B) Heckmann, and (C) Chen data sets. The plots also include the AUC and AUPR statistics.

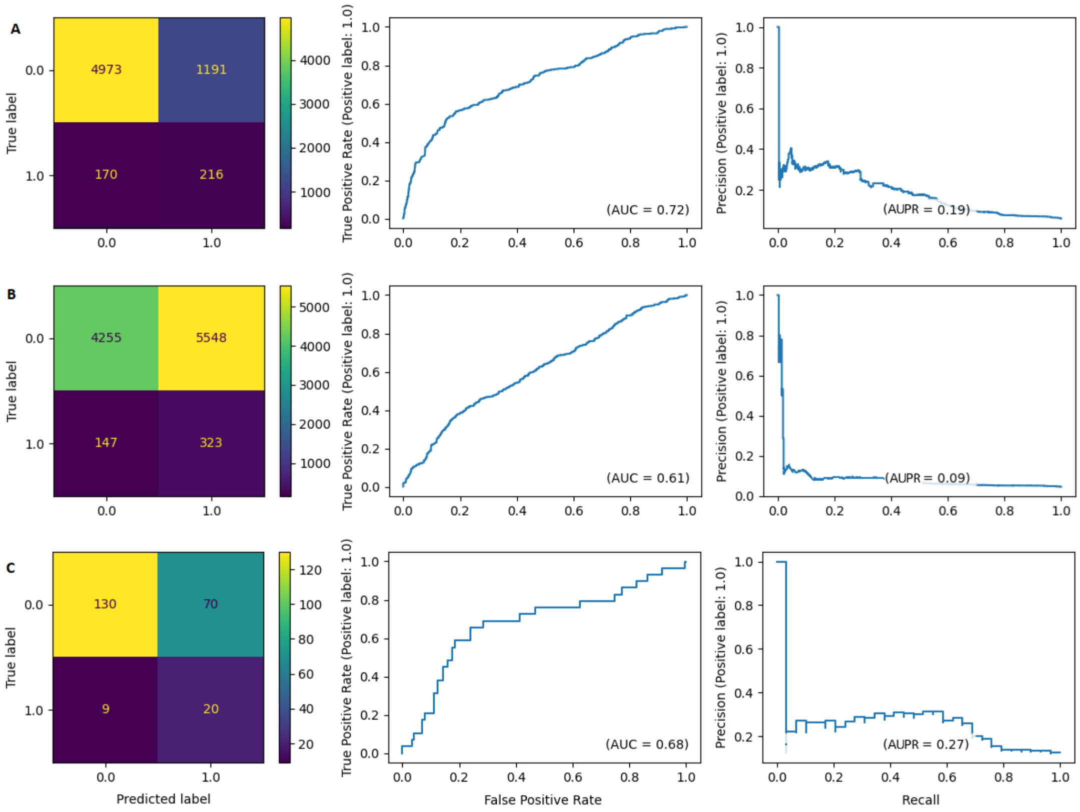

Figure S5. Performance of the MLP classifier on three data sets using the random STITCH labeling strategy to label negative instances. The plots show the confusion matrices alongside the ROC and PR (Precision/Recall) curves for the classifiers trained with the featured obtained based on the (A) Davidi, (B) Heckmann, and (C) Chen data sets. The plots also include the AUC and AUPR statistics.

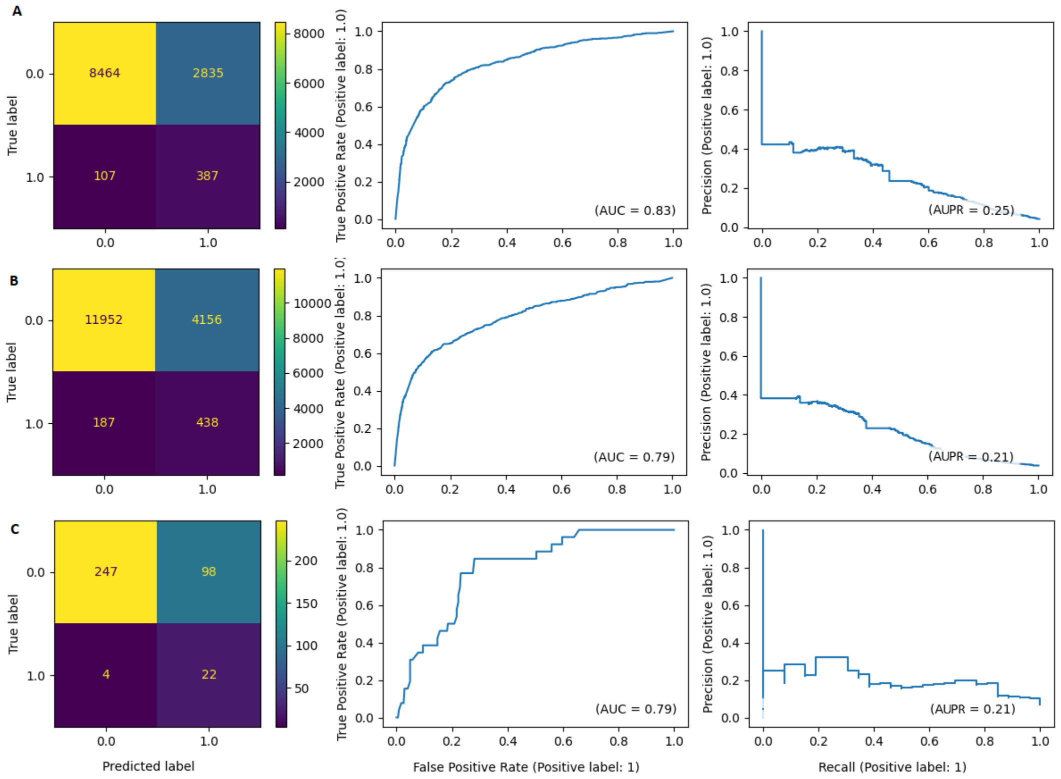

Figure S6. Performance of the RF classifier on three data sets using the Tanimoto labeling strategy to label negative instances. The plots show the confusion matrices alongside the ROC and PR (Precision/Recall) curves for the classifiers trained with the featured obtained based on the (A) Davidi, (B) Heckmann, and (C) Chen data sets. The plots also include the AUC and AUPR statistics.

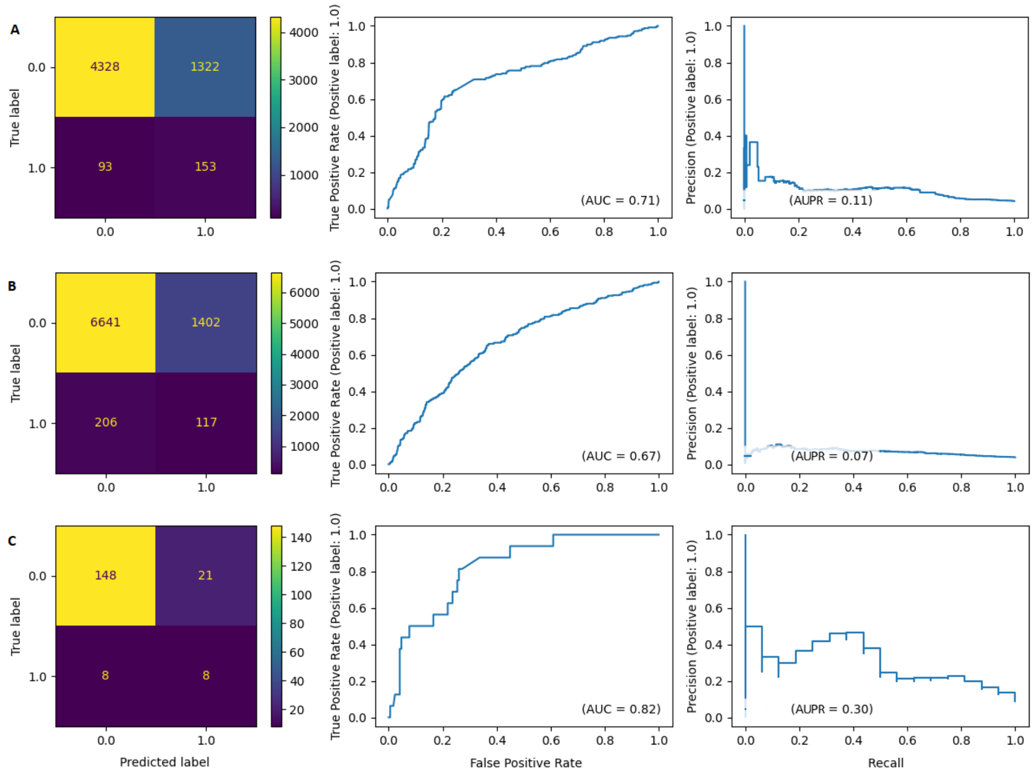

Figure S7. Performance of the SVM classifier on three data sets using the Tanimoto labeling strategy to label negative instances. The plots show the confusion matrices alongside the ROC and PR (Precision/Recall) curves for the classifiers trained with the featured obtained based on the (A) Davidi, (B) Heckmann, and (C) Chen data sets. The plots also include the AUC and AUPR statistics.

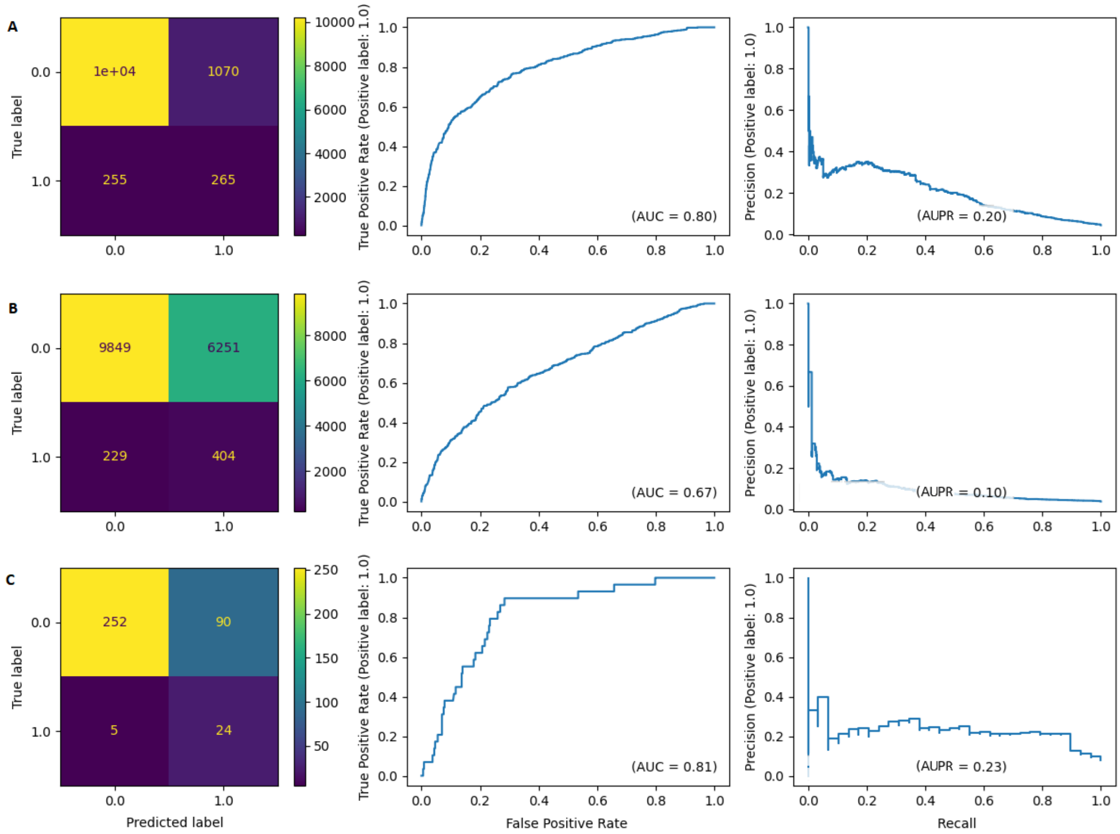

Figure S8. Performance of the MLP classifier on three data sets using the Tanimoto labeling strategy to label negative instances. The plots show the confusion matrices alongside the ROC and PR (Precision/Recall) curves for the classifiers trained with the featured obtained based on the (A) Davidi, (B) Heckmann, and (C) Chen data sets. The plots also include the AUC and AUPR statistics.

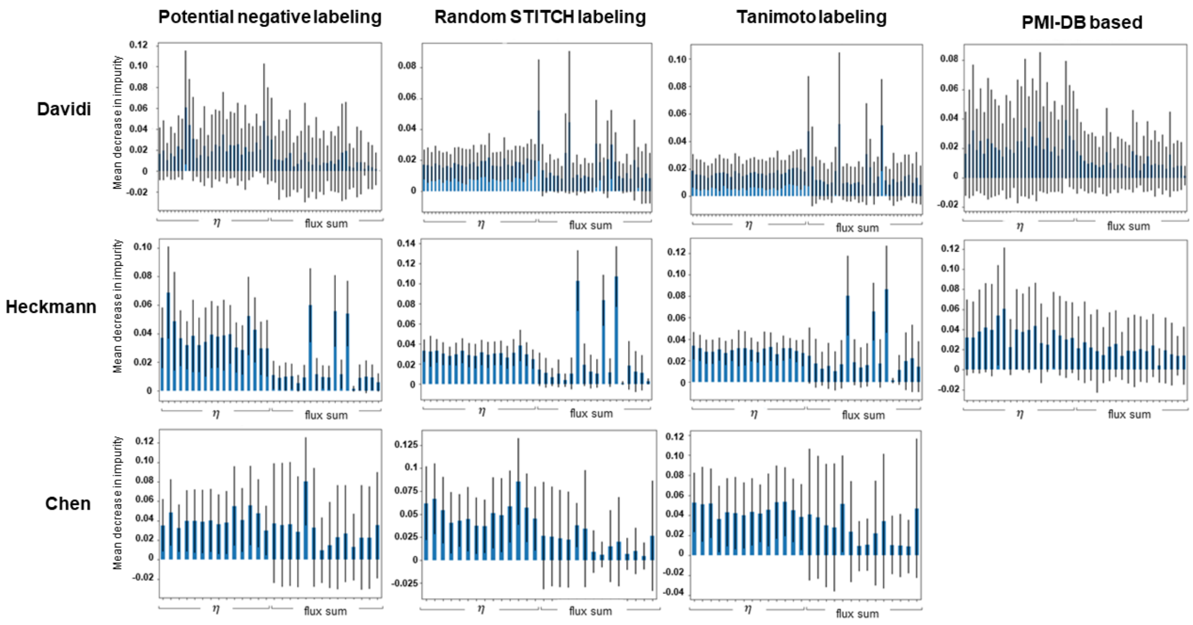

Figure S9. Feature importance analysis of the RF classifiers built on the concatenated features for predicting MPIs. The RF classifiers are trained on the concatenated features derived from η values and flux sums across three examined data sets using different negative labeling strategies. The plots show the mean decrease in impurity across different data sets and labeling strategies, highlighting the importance of the features in MPI prediction.
